# Vascular cognitive impairment and dementia: a narrative review

**DOI:** 10.1590/1980-5764-DN-2023-0116

**Published:** 2024-09-23

**Authors:** Amado Jiménez-Ruiz, Victor Aguilar-Fuentes, Naomi Nazareth Becerra-Aguiar, Ivan Roque-Sanchez, Jose Luis Ruiz-Sandoval

**Affiliations:** 1 Stroke & Cerebrovascular Disease Clinic, Hospital Civil Fray Antonio Alcalde, Neurology Department, Guadalajara, Jalisco, Mexico.; 2 Universidad de Guadalajara, Centro Universitario de Ciencias de la Salud, Departamento de Neurociencias, Guadalajara, Jalisco, Mexico.

**Keywords:** Vascular Dementia, Cognition Disorders, Dementia, Cerebrovascular Diseases, Stroke, Demência Vascular, Transtornos Cognitivos, Demência, Transtornos Cerebrovasculares, Acidente Vascular Cerebral

## Abstract

Vascular cognitive impairment (VCI) is the second most common cause of cognitive impairment after Alzheimer's disease. The VCI spectrum involves a decline in cognition attributable to vascular pathologies (e.g., large infarcts or hemorrhages, microinfarcts, microbleeds, lacunar infarcts, white matter hyperintensities, and perivascular space dilation). Pathophysiological mechanisms include direct tissue injury, small vessel disease, inflammaging (inflammation + aging), atrophy, and altered neurotransmission. VCI is diagnosed using distinct clinical and radiological criteria. It may lead to long-term disability and reduced quality of life. An essential factor for reducing cognitive impairment incidence is preventing stroke by managing traditional and non-traditional cerebrovascular risk factors. This article reviews the spectrum of VCI, epidemiology, risk factors, pathophysiology, diagnosis, available treatment, and preventive strategies.

## RESUMO

O comprometimento cognitivo vascular (CCV) é a segunda causa mais comum de comprometimento cognitivo depois da doença de Alzheimer. O espectro do CCV envolve um declínio na cognição atribuível a patologias vasculares (por exemplo, grandes infartos ou hemorragias, microinfartos, micro-hemorragias, infartos lacunares, hiperintensidades da substância branca e dilatação do espaço perivascular). Os mecanismos fisiopatológicos incluem lesão tecidual direta, doença de pequenos vasos, *inflammaging* (inflamação+envelhecimento), atrofia e neurotransmissão alterada. O CCV é diagnosticado usando critérios clínicos e radiológicos distintos. Pode levar à incapacidade a longo prazo e à redução da qualidade de vida. Um fator essencial para reduzir a incidência de comprometimento cognitivo é prevenir o acidente vascular cerebral através do manejo dos fatores de risco cerebrovasculares tradicionais e não tradicionais. Este artigo revisa o espectro do CCV, epidemiologia, fatores de risco, fisiopatologia, diagnóstico, tratamento disponível e estratégias preventivas.

## INTRODUCTION

Cognition is one of the primary brain functions and plays a vital role in the aging process. Cognitive impairment leads to increased dependency and decreased quality of life throughout the lifespan. Assuring adequate cognition and acknowledging its detrimental factors, such as cardiovascular diseases, may prevent cognitive impairment and lead to a healthier lifestyle and better brain health^1^.

Acquiring, processing, storing, and retrieving information by the brain refers to normal cognition composed of memory, executive functioning, perception, attention, motor movement, and language skills^2^. An impairment in cognition is classified into a spectrum of disorders ranging from mild to severe, including age-related cognitive decline, mild cognitive impairment, and major cognitive impairment^3^.

According to the Diagnostic and Statistical Manual of Mental Disorders fifth edition (DSM-V), mild neurocognitive disorder refers to a decline in one or more cognitive domains (executive function, learning, memory, complex attention, language, social cognition, and perceptual-motor) without interfering with independence in everyday activities. Major neurocognitive disorder (previously known as dementia) involves cognitive deficits that interfere with independence in daily activities. Both conditions can be further classified according to etiology (Alzheimer's disease, frontotemporal lobar degeneration, Lewy body dementia, Parkinson's disease, vascular, and related, among others). The second most common cause of neurocognitive disorder is vascular in origin, a condition known as vascular cognitive impairment (VCI)^4^.

VCI refers to cognitive decline attributable to cerebrovascular disease, including all forms of stroke. Recent developments have changed our understanding of this complex syndrome affecting millions of individuals worldwide. Here, we briefly review the currently available evidence and explore the associated challenges with diagnosis and treatment.

## THE SPECTRUM OF VASCULAR COGNITIVE IMPAIRMENT

VCI involves a spectrum of vascular brain pathologies contributing to any degree of cognitive impairment^5,6^. According to the Vascular Impairment of Cognition Classification Consensus Study phase 2 (VICCCS-2), VCI can be classified into mild VCI (VaMCI) or major VCI (vascular dementia, VaD) depending on the severity and the compromise of the ability to perform activities of daily living. VaD is further subclassified as poststroke dementia, subcortical ischemic vascular dementia, multi-infarct dementia, and mixed dementia^7^. Classification criteria will be further discussed.

## EPIDEMIOLOGY OF COGNITIVE IMPAIRMENT

MCI is a syndrome in which patients have cognitive decline with minimal impairment in their activities of daily living. Its prevalence varies according to age, ranging from 6.7% for individuals aged 60–64 years to 25.2% for those aged 80–84^8^. The incidence increases with age, and more than half of individuals with MCI progress to dementia within five years after diagnosis^9^. MCI prevalence varies according to the population and risk factors. Depression, diabetes, hypertension, rurality, and low education are associated with increased risk of MCI^10^. In Latin America, it ranges from 6.8 to 25.5%, with a higher prevalence in the oldest, lower-educated adults, women, and rural settings^11,12^.

Approximately 50 million people live with dementia worldwide, estimated to increase to 152 million by 2050. Dementia is decreasing in developed countries but increasing in developing countries. Approximately 66.0% of individuals with dementia live in low- and middle-income countries, but by the year 2050, it is projected to increase to 71.0 or 72.0%^13^. In Brazil, approximately 1.5 million people live with dementia with an estimated prevalence of 17.5%^14^. Over the last years, the burden of dementia has been steadily rising in Brazil, being the second cause of death by 2016^15^. In the Mexican population, the estimated prevalence of dementia is 7.8% for Alzheimer's disease (AD), 4.3% for VaD, and 2.1% for mixed dementia^16^. However, in a clinical-pathological analysis from two longitudinal studies (The Religious Orders Study and the Rush Memory and Aging Project), only 9.0% had AD, whereas 40.0% consisted of AD plus prominent vascular pathologies such as macroscopic infarcts, cerebral amyloid angiopathy, atherosclerosis, or arteriosclerosis^17^. A Mexican cohort study estimating the incidence of cognitive impairment in previously cognitively healthy Mexican individuals revealed that 20.0% of the subjects developed moderate cognitive impairment, of which 10.0% developed severe cognitive impairment during a three-year follow-up^18^. Similarly, Argentina and Venezuela, other Latin American countries, presented a prevalence of dementia of 7.8% and 8.04%, respectively, mostly attributed to physical inactivity, hypertension, obesity, and hearing loss^19-21^.

## EPIDEMIOLOGY OF VASCULAR COGNITIVE IMPAIRMENT

The prevalence of VCI has varied across the years due to the different diagnostic criteria and definitions implemented.

VCI is considered the second most common etiology of dementia. A neuropathological study revealed that in a population of 407 individuals with MCI, 23.0% showed a pure vascular neuropathological result, whereas 51.0% had a mixed neuropathological result. In patients with dementia, only 7.0% had a pure vascular neuropathological result, and 70.0% had a mixed neuropathological result^22^.

Post-stroke cognitive impairment (PSCI) is a common cause of long-term disability and reduced quality of life^23^. Previous studies in European populations indicated that in the first year after the stroke, half of the patients may develop some degree of cognitive impairment^24,25^. The incidence of PSCI in Latin America has not been fully addressed. In a Mexican study, PSCI was presented in up to 41.0% of patients^26^. However, a Brazilian study showed contrasting results with a PSCI incidence of 16.8%^27^. The difference may be due to the population included—while the Mexican study considered cerebral venous thrombosis, and both ischemic and hemorrhagic stroke, the Brazilian study focused mainly on non-embolic ischemic stroke patients.

## RISK FACTORS OF VASCULAR COGNITIVE IMPAIRMENT

The incidence and prevalence of dementia have decreased in developed countries and increased in developing countries; this phenomenon is mainly attributed to reduced cerebrovascular risk factors, such as hypercholesterolemia and smoking in developed countries, which are highly associated with VCI^13^.

**Figure 1:**
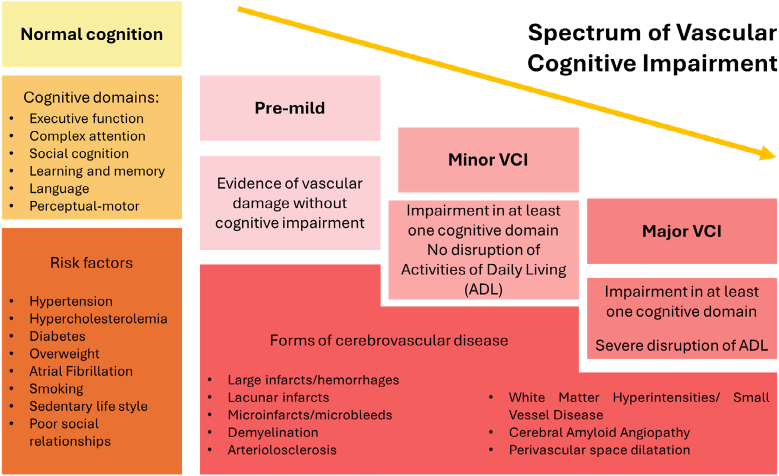
The spectrum of vascular cognitive impairment. Progression and features in different stages of cognitive decline.

VCI is a stepwise process in which many diseases and sociodemographic conditions may contribute (Figure 1). Some common risk factors for VCI include diabetes mellitus, overweight, obesity, atrial fibrillation, elevated low-density lipoprotein, and aging (especially males >80 years old)^6,28^. Hypertension and depression are strong predictors of possible VaD^29^. Poor social relationships and deficient support networks are also associated with white matter hyperintensity (WMH) progression, another biomarker of cognitive impairment^30^. Individuals with a stroke have a 9-fold increased risk of developing cognitive impairment in the first year after the event^31^. This risk increases with recurrent strokes^32^.

Approximately 40.0% of dementia risk factors are potentially modifiable, most related to cardiovascular damage^33^. This means that by preventing cardiovascular and metabolic diseases, many dementia cases may be preventable. Sposato et al. proposed, from a retrospective analysis of the incidence rate of dementia and stroke from a 10-year Canadian survey, that the reduction in stroke by 32.4% could have contributed to the 7.4% reduction in dementia incidence^34^.

## PATHOPHYSIOLOGY

Researchers previously associated cognitive impairment with large vessel arteriosclerosis, a condition known as arteriosclerotic dementia^35^. Although incomplete, this hypothesis was not far from the current mechanisms involved in VCI, making brain hypoperfusion a fundamental pathophysiological mechanism^36^. Nevertheless, arteriosclerosis is not the only process involved in this complex syndrome. Other processes that contribute to VCI presentation are large infarct, lacunar infarct, microinfarct, myelin loss, WMH, cerebral amyloid angiopathy, and perivascular space dilation^37^.

**Figure 2:**
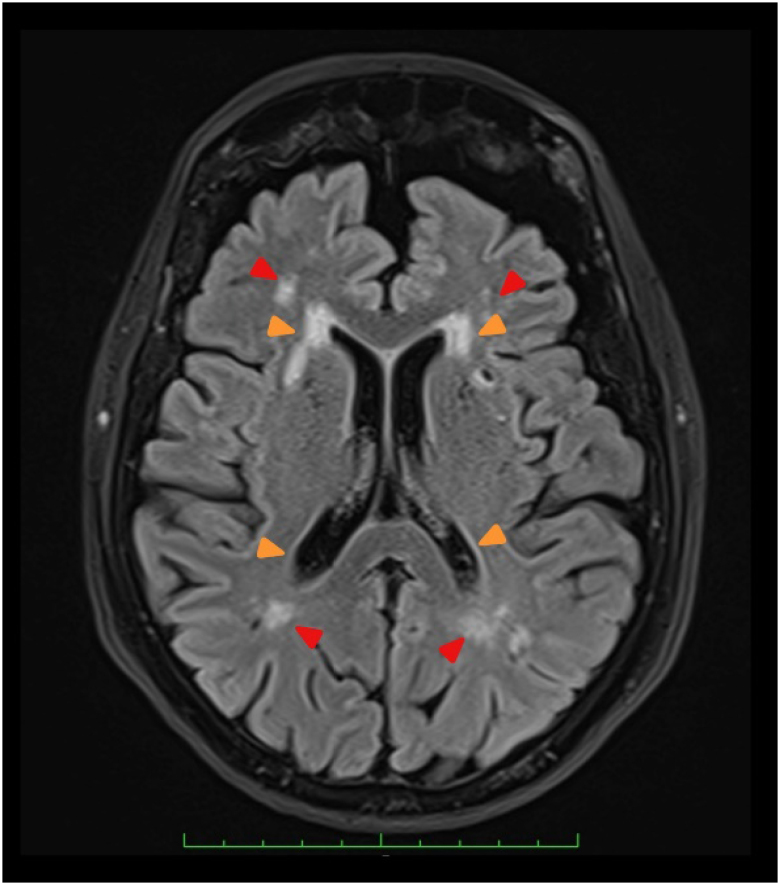
An 81-year-old woman with cognitive impairment. Brain magnetic resonance imaging in fluid-attenuated inversion recovery (FLAIR) sequence (axial view) showing subcortical (red arrowhead) and periventricular (orange arrowhead) white matter hyperintensities consistent with small vessel disease. Global brain atrophy is also observed (personal communication).

**Figure 3:**
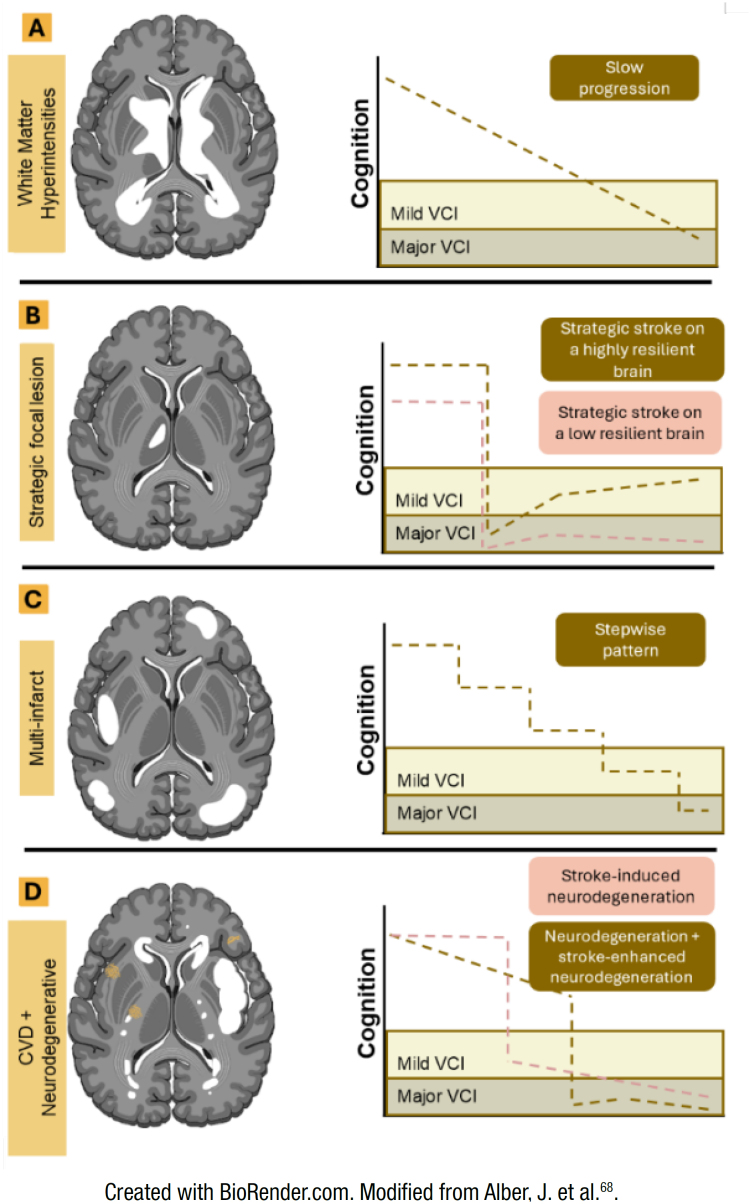
Distinctive phenotypes of cognitive decline with corresponding vascular pathology. (A) White matter hyperintensities have a slow progression of cognitive decline. (B) Strategic focal lesion has an acute one-step pattern with either some recovery or decline progression depending on the brain resilience. (C) Multi-infarct dementia is consistent with a stepwise pattern of vascular cognitive impairment (VCI). (D) Cerebrovascular disease (CVD) + neurodegenerative (mixed mechanism) may present in two sceneries: the first is an already damaged brain by a degenerative disease that suffered a brain infarction with subsequent cognitive decline; and the second is a one-step pattern that triggers a neurodegenerative process. Cerebral infarcts and white matter disease are shown in white. Amyloid plaques are indicated in orange.

VCI may present in a slow and progressive matter, such as seen in small vessel disease (Figure 2) and multi-infarct dementia (Figures 3A and 3C), or in a sudden matter, such as seen in a large strategic infarct (Figure 3B). Stroke may also trigger cognitive impairment in an already damaged brain (Figure 3D).

Slowly progressive VCI is usually seen in small vessel disease (SVD). The most common subtype of SVD is cerebral amyloid angiopathy, a condition also associated with intracranial hemorrhage, microbleeds, and microinfarcts. SVD leads to VCI via hypoperfusion, ischemic neuronal injury, and diffuse tissue injury^6^. Subcortical dementia (previously known as Binswanger disease) is a form of SVD associated with damage mainly in the white matter; the injury is conformed by gradual subcortical ischemia and WMH^38,39^.

SVD and subsequent VCI may also be a product of genetic disturbances. Cerebral autosomal dominant arteriopathy with subcortical infarcts and leukoencephalopathy (CADASIL) is the most common cause of genetic SVD; its early identification and management can increase the quality of life in this progressive and irreversible condition^40^. Vascular smooth muscle cell degeneration, seen in CADASIL, results in an impaired muscular layer of brain vessels. This impairment leads to vessel breakdown, fibrosis, and thickening of the vessels with subsequent stenosis, hypoperfusion, and brain infarcts^41^. Genetic causes of SVD present cognitive impairment early in life and are the purest types of VCI.

Aging is another slow and progressive mechanism that stimulates multiple vascular changes, starting with the thinning of large and small blood vessels. A decrease in cerebral blood flow contributes to a reduction in glucose uptake, an increase in collagen accumulation, a rupture of the blood-brain barrier, decreased tight junctions, decreased mitochondrial contents, inflammation, and oxidative damage. These processes contribute to neuron loss and increased susceptibility to cognitive decline^42^.

Large strategically located infarcts mainly cause sudden onset VCI. The most associated locations are the thalamus, the hippocampus, and the dominant hemisphere^6,32,43^. Although strategically located infarcts are highly associated with cognitive impairment, it is essential to know that the manifestation of cognitive impairment also depends on cognitive reserve. This concept will be discussed later on.

Other brain insults leading to VCI are macroscopic hemorrhages, microbleeds, and microinfarcts. Microinfarcts are only observable using microscopy due to their small diameters of 0.2 to 1 mm. Although the mechanism of dementia is still unclear, it may be associated with innate and adaptive immune responses and impaired protein clearance^6^.

An important subtype of VCI is post-stroke dementia (PSD)^44,45^. This condition refers to VaD detected after an ischemic or hemorrhagic stroke. PSD can be further subclassified into early-onset PSD and late-onset PSD. Early-onset PSD is a condition in which cognitive impairment is frequently detected in the first six months after a stroke. It is usually driven by cardiovascular risk factors (hypertension, atrial fibrillation, hyperlipidemia, and diabetes) and stroke lesion characteristics (location and size). Early-onset PSD appearance also depends on brain resilience. Brain resilience, which is constituted by cognitive reserve and brain reserve, refers to the capacity of the brain to resist and recover its regular functions after an insult. More brain resilience is associated with lower odds of developing early-onset PSD. Late-onset PSD affects up to 23.9% of individuals with a previous stroke^32^. The presentation time of this condition is variable but may occur up to eight years after the brain insult^46^, augmenting the need for continuous cognitive screening throughout the lifespan. Late-onset PSD is caused by recurrent strokes, amyloid-beta deposition, and small vessel disease, the last being the most important etiological factor driving this incapacitating disease^32^. Severe WMH is associated with a 7.7-fold increased risk of developing severe cognitive impairment^47^. The amyloid β deposition is known to be related to dementia incidence; however, little is known about the role of ischemia in amyloid β deposition. Cerebral hypoxia reduces the activity of lipoprotein receptor-related protein 1 (LRP1) and boosts the activity of receptor for advanced glycation end products (RAGE), β-secretase, and γ-secretase^36,48^. Thereby, it increases the production and accumulation and decreases the clearance of amyloid β. Amyloid β deposition further decreases brain perfusion by inducing vasoconstriction. Both of these mechanisms work in a vicious cycle that culminates in an elevated risk of developing dementia^49-51^.

Other mechanisms involved in VCI are local and systemic inflammation, neuronal atrophy, oligodendrocyte and astrocyte changes, and altered signaling due to impaired lymphatic clearance of toxins and proteins^6,52^.

## CLINICAL MANIFESTATIONS

Typically, patients with VCI have mental slowness and impaired executive function (cognitive flexibility, planning, organizing, inhibition, and self-regulation). Cognitive impairment affecting only executive functions (non-amnestic cognitive impairment) is more frequent in VCI than in AD and can present in up to 49.0% of individuals. However, VCI individuals may also exhibit memory disturbances, especially if there is damage to the medial temporal lobe, in which case, there is a 2.7-fold increased risk of developing amnestic VCI^53^. Increased amyloid β deposition may also contribute to the development of amnestic VCI.

Memory loss and behavioral and psychiatric symptoms, including apathy, anxiety, and depression, are frequent and should also be pursued when evaluating an individual with VCI^6^.

## DIAGNOSIS

VCI is a complex term that has evolved over the years. To have a clearer understanding of the concept and its diagnosis, it is essential to quickly recap the evolution of this term.

At first, cognitive impairment was related to large vessel arteriosclerosis (arteriosclerotic dementia). However, this idea was only partially accepted^35^. Later, amyloid plaques were considered as the sole etiology of cognitive impairment (Alzheimerization of dementia). After many years, Hachinski et al. introduced the term "multi-infarct dementia" to refer to individuals with slow and progressive major cognitive impairment, primarily related to cardioembolic causes^54^. This term persists nowadays. Nevertheless, it is known that cardioembolism is not the sole mechanism involved in VCI. Therefore, the National Institute of Neurological Disorders and Stroke (NINDS) and the *Association Internationale pour la Recherche et L’Enseignement en Neurosciences* (AIREN, International Association for Research and Education in Neuroscience) proposed the diagnostic criteria for VaD to incorporate other etiologies of cognitive impairment related to vascular disease^55,56^.

An inconvenience with these diagnostic criteria was that the cognitive evaluation was mainly focused on memory impairment. Although memory impairment is seen in individuals with VCI, the majority, especially in the early stages, present cognitive impairment mainly in executive functions. PSD is a term that refers to individuals with major cognitive impairment detected after a stroke^44,45^. This definition was greatly accepted and is still used in most reviews. However, this term did not distinguish between pure vascular PSD and mixed PSD. This led to an overdiagnosis of dementia related to cardiovascular diseases, which was corrected with more recent diagnostic criteria^7^.

After many years of studying major cognitive impairment related to vascular diseases, an inconvenience remained: late diagnosis. Individuals with VaD, by definition, already had an established severe impairment in the activities of daily living. Therefore, VCI was introduced to detect individuals with cognitive impairment related to cardiovascular diseases and, if possible, prevent major cognitive dysfunction^57^.

**Figure 4:**
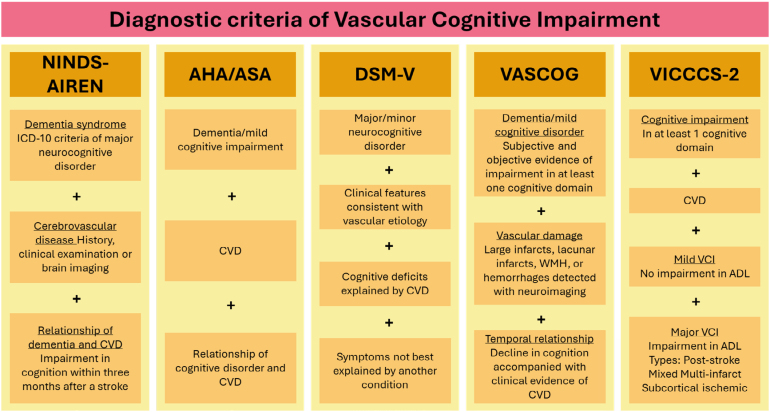
Diagnostic criteria of vascular cognitive impairment. Summary of the proposed diagnostic criteria for vascular cognitive impairment proposed by the National Institute of Neurological Disorders and Stroke and the *Association Internationale pour la Recherche et l’Enseignement en Neurosciences*, the American Heart Association/The American Stroke Association; the Diagnostic and Statistical Manual of Mental Disorders fifth edition, the International Society for Vascular Behavioral and Cognitive Disorders, and the Vascular Impairment of Cognition Classification Consensus Study phase 2.

Abbreviations: NINDS, National Institute of Neurological Disorders and Stroke; AIREN, Association Internationale pour la Recherche et l’Enseignement en Neurosciences; AHA, American Heart Association; ASA, American Stroke Association; DSM, Diagnostic and Statistical Manual of Mental Disorders; VASCOG, Vascular Behavioral and Cognitive Disorders; VICCCS, Vascular Impairment of Cognition Classification Consensus Study; ICD, international classification of diseases; CVD, cerebrovascular disease; ADL, activities of daily living; WMH, white matter hyperintensities.

Afterward, other diagnostic criteria were introduced to define minor and major cognitive impairment related to vascular diseases (Figure 4). The most used diagnostic criteria are The American Heart Association/The American Stroke Association (AHA/ASA), which define vascular mild cognitive impairment (VaMCI) and VaD, both subdivided into probable and possible^58,59^; the DSM-V, which defines minor and major neurocognitive disorder^4^; The International Society for Vascular Behavioral and Cognitive Disorders (VASCOG), which classifies mild and major vascular cognitive disorders^60,61^; and the VICCCS-2, which distinguishes between mild VCI and VaD^7^. Some national academies, such as the Brazilian Academy of Neurology, also includes their own criteria with great reliability^62^.

VICCCS-2 diagnostic criteria remain the most suggested for determining VCI. The preferred imaging method for diagnosing cerebrovascular brain injury according to the VICCCS-2 criteria is magnetic resonance imaging (MRI)-based with measures of the number, size, locations of infarcts and hemorrhages, total brain volume (including the hippocampus), and WMH burden^7^.

Mild and major cognitive impairment are the two major subcategories of VCI, according to the VICCCS-2 diagnostic criteria, with the main difference regarding the severe disruption of activities of daily living. Major cognitive impairment is further subdivided into post-stroke dementia (history of stroke and cognitive deficit either immediately after or within six months after the insult and no recovery), subcortical ischemic vascular dementia (small-vessel disease mainly subcortically), multiinfarct (cortical) dementia, and mixed dementias (combination of vascular and degenerative disease)^61^.

Pre-mild VCI or "brain at risk" stage is discussed as a stage previous to cognitive impairment, in which there is no cognitive impairment, but there is evidence of vascular damage, which may be a future VCI risk^63^.

Assessing cognitive impairment in patients with a cerebrovascular etiology is challenging since the cognitive batteries are biased toward AD, and the cognitive domains affected in VCI are different, with a preponderance of executive function. Among the first tools to assess cognitive deficits in patients with cerebrovascular disease is the cognitive impairment harmonization standards created by NINDS - Canadian Stroke Network (NINDS-CSN). The protocols are divided into very short (5 minutes), short (30 minutes), and extensive (1 hour). The tests used include Animal Naming, Trail Making, Rey-Osterrieth Complex, Hopkins Verbal Learning, and Naming Test Recognition, among others^64^. Since then, adaptations to the NINDS-CSN have been proposed such as the transitional and final battery of the *Groupe de Réflexion pour l’Evaluation COGnitive VASCulaire* (GRECog-Vasc, Reflection Group for Vascular Cognitive Evaluation) adapted for the French population^65^.

Apart from the NINDS-CSN criteria, the most widely validated tools are the Clock Drawing, the Montreal Cognitive Assessment (MoCA), the Mini-Mental State Examination (MMSE), and the Brief Memory and Executive Test (BMET), tools that have been compared and evaluated in different studies. Results have reported that MoCA applied 3–6 months after a stroke is more sensitive and superior to the NINDS-CSN 5-minute protocol^66,67^. A systematic review evaluating the psychometric properties of cognitive screening instruments in VCI revealed that the MoCA (92.7% sensitivity, and 96.3% specificity) and MMSE (78.0% sensitivity, and 92.0% specificity) had excellent accuracy in differentiating VaD from controls, and the MoCA and BMET had the greatest accuracy in separating mild VCI from controls (sensitivity and specificity of 81.0 and 79.0, and 85.0 and 84.0%, respectively). The study concluded that the MoCA is accurate and reliable for differentiating VaD and mild VCI^67^.

The other aspect of the diagnosis involves establishing the vascular etiology for the cognitive disorder with neuroimaging evidence (preferably with MRI) of large infarcts, lacunar infarcts, WMH, or hemorrhages, as well as a temporal relationship with the decline in cognition accompanied by personality and mood changes, hemiparesis, lower facial weakness, visual field defects, sensory loss, gait disturbance, and urinary symptoms consistent with clinical evidence of cerebrovascular disease^60^.

## TREATMENT

The non-pharmacological management of VCI includes dietary interventions and exercise. Several studies suggest the consumption of omega-3 fatty acids, non-fried fish, and the Mediterranean diet to avoid WMH progression and cognitive decline^68^. A randomized controlled trial evaluated the effect of progressive aerobic exercise training on cognitive and executive functions and concluded that the intervention group performed better on cognitive scales^58^. Cheon et al. demonstrated that exercise reduces the risk of developing PSD^69^.

An interventional study evaluating the progression of WMH in people with diabetes using a multidomain approach concluded that lifestyle interventions such as physical activity and diet modifications reduced the WMH burden. However, there were no differences in cognitive function compared to the control group^70^.

The pharmacological treatment of VCI involves cholinesterase inhibitors such as donepezil, rivastigmine, and galantamine, commonly used for AD. According to the AHA/ASA recommendations and recent meta-analyses, donepezil and galantamine have the most significant effect on cognition^59,71^; however, these results are insufficient to recommend for routine use and are not currently approved by the US Food and Drug Administration. In other studies, rivastigmine showed a slight improvement in executive function and behavior, and memantine resulted in a slight improvement in cognition^72,73^. Another group of drugs that may prevent dementia progression includes anticoagulants^74^. Recent meta-analyses and population-based studies support the hypothesis that anticoagulants (direct oral anticoagulants and vitamin K inhibitors) may prevent cognitive impairment^75-77^. Anecdotal evidence supporting anticoagulant use for preventing dementia progression has been available since 1968^74^.

As mentioned above, atrial fibrillation is related to the development of multiple cardioembolic strokes, making it an important risk factor for the development of VCI. Even though anticoagulation is generally recommended, the choice of anticoagulant is not fully elucidated. A Brazilian randomized clinical trial (GIRAF, CoGnitive Impairment Related to Atrial Fibrillation) evaluated the cognitive outcomes of dabigatran versus warfarin in older patients with atrial fibrillation, revealing no statistically significant difference in changes in cognitive performance between both anticoagulant strategies; therefore, there is no evidence of a beneficial effect of dabigatran^78^.

To avoid stroke recurrence, a strong risk factor for cognitive decline, the AHA/ASA guidelines recommend antihypertensives such as diuretics and angiotensin-converting enzyme inhibitors (ACEi) if blood pressure is ≥140 mmHg systolic or ≥90 mmHg diastolic. Other recommended medications are lipid-lowering drugs, especially statins, antithrombotics, and anticoagulants for cardioembolic stroke or transient ischemic attack^79^. Moreover, treatment to prevent the progression of WMH includes antihypertensives, such as ACEi and angiotensin receptor blockers, statins, and antithrombotics, including antiplatelet drugs like aspirin^68^.

Pharmacological strategies are the cornerstone for treating VCI, but rehabilitation may also improve cognition. A randomized active-controlled clinical trial showed improved global cognitive function and better results in the MoCA test in the group of people with subcortical VCI taking a 7-week computerized cognitive training course; the study also revealed increased functional connectivity between the left dorsolateral prefrontal cortex and medial prefrontal cortex after training^80^. Other cognitive rehabilitation programs have evidenced improvement in specific domains, such as working memory and attention and synchronization of activity in cerebellar areas^81^.

## PREVENTION

Hachinski et al. proposed preventing dementia (at least one-third of them) through stroke prevention, according to the Berlin Manifesto. Stroke and dementia are risk factors for each other and share some of the same protective factors, such as the reduced prevalence of smoking, anticoagulation for atrial fibrillation, and systolic blood pressure lower than 140 mmHg^82^.

A retrospective cohort study evaluating the effect of thrombolysis on the incidence of poststroke dementia showed that thrombolysis administration within three hours of the stroke was associated with a decreased rate (24.0% at one year and 21.0% at five years) of developing dementia^83^.

VCI has known modifiable risk factors, and impairment in cognition may be prevented by managing them^84^. Optimal blood pressure, blood glucose, total cholesterol, smoking habits, physical activity, body mass index, healthy diet, and cognitively and socially stimulating activities are metrics for brain health, which are essential for cognition preservation^85^. Although only two of these metrics have strong evidence for prevention-risk patients (hypertension control and physical activity), they are reasonable strategies for overall mortality and vascular disease prevention^59^.

In the Systolic Blood Pressure Intervention Trial — Memory and Cognition in Decreased Hypertension (SPRINT MIND) study, 9,361 older adults with some cardiovascular risk factors but no diabetes, stroke, or dementia were followed up for one year, evaluating progression to mild cognitive impairment and dementia. There was a statistically significant lower rate of mild cognitive impairment progression and non-statistically significant dementia progression in the intensive (<120 mm Hg systolic pressure) vs. the standard treatment group (<140 mm Hg systolic pressure)^86^.

The Finnish Geriatric Intervention Study to Prevent Cognitive Impairment and Disability (FINGER) multimodal intervention (diet, exercise, cognitive training, and vascular risk monitoring) provided strong evidence for preventing AD and VCI, and it seems promising as an essential brain health strategy^87^.

New studies are evaluating the effect of oral anticoagulants and vitamin K inhibitors on reducing cognitive decline and dementia risk. There is also interest in a specific group of patients with non-valvular atrial fibrillation. In patients with cerebral small vessel disease, there is evidence of increased bleeding risk and no effect on cognitive function. Few studies limit the recommendations regarding oral anticoagulants and vitamin K inhibitors as preventive measures^75,88,89^.

VCI is a highly heterogeneous disease. Recent evidence has helped us improve our understanding of how cerebrovascular disease contributes to cognitive impairment.

More research is needed to explain the relationship between VCI and neurodegenerative disease. Preventing vascular injury by treating modifiable risk factors remains a cornerstone in the fight against cognitive impairment. More studies are needed to develop symptomatic and disease-modifying treatments.
